# Modified Smoluchowski Rate Equations for Aggregation and Fragmentation in Finite Systems

**DOI:** 10.1021/acs.jpcb.3c02884

**Published:** 2023-06-27

**Authors:** Beata Szała-Mendyk, Aleksandra Drajkowska, Andrzej Molski

**Affiliations:** Faculty of Chemistry, Adam Mickiewicz University in Poznań, Uniwersytetu Poznańskiego 8, 61-614 Poznań, Poland

## Abstract

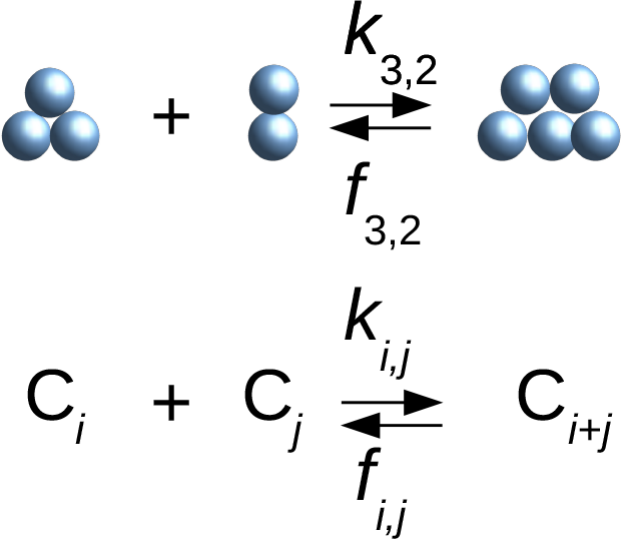

Protein self-assembly into supramolecular structures is important for cell biology. Theoretical methods employed to investigate protein aggregation and analogous processes include molecular dynamics simulations, stochastic models, and deterministic rate equations based on the mass-action law. In molecular dynamics simulations, the computation cost limits the system size, simulation length, and number of simulation repeats. Therefore, it is of practical interest to develop new methods for the kinetic analysis of simulations. In this work we consider the Smoluchowski rate equations modified to account for reversible aggregation in finite systems. We present several examples and argue that the modified Smoluchowski equations combined with Monte Carlo simulations of the corresponding master equation provide an effective tool for developing kinetic models of peptide aggregation in molecular dynamics simulations.

## 1 Introduction

Protein self-assembly into supramolecular structures is important for cell biology,^1^ medicine,^2^ and materials science.^3^ Theoretical methods employed to investigate protein aggregation and analogous processes include molecular dynamics simulations,^4,5^ stochastic models,^6−12^ and deterministic rate equations based on the mass-action law.^13−16^

The Smoluchowski coagulation model is a deterministic theory that describes irreversible aggregation.^17,18^ This model assumes that the state of the system is specified by the discrete distribution of cluster sizes *c*_*i*_(*t*), where *c*_*i*_(*t*) denotes the instantaneous concentration of clusters C_*i*_ of size *i* at time *t*. The time evolution of the cluster size distribution, *c*_*i*_(*t*), is described by a set of rate equations:
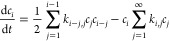
1where *k*_*i*,*j*_ are the second-order rate constants of bimolecular aggregation between clusters of sizes *i* and *j*. The first term on the right-hand side (rhs) is the rate of creation of clusters C_*i*_ through aggregation of smaller clusters, C_*j*_ + C_*i*–*j*_ → C_*i*_. The second term is the rate of decay of clusters C_*i*_ caused by aggregation, C_*i*_ + C_*j*_ → C_*i*+*j*_. The Smoluchowski deterministic theory can be extended to allow for reversible aggregation:^18,19^
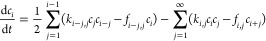
2where *f*_*i*,*j*_ are the first-order rate constants for the monomolecular fragmentation of clusters C_*i*+*j*_ into clusters C_*i*_ and C_*j*_, C_*i*+*j*_ → C_*i*_ + C_*j*_. The first two terms on the rhs are the rates of creation and decay of clusters C_*i*_ through aggregation of smaller clusters and fragmentation into smaller clusters, respectively. The third term is the rate of decay of clusters C_*i*_ by aggregation. The fourth term is the rate of creation of clusters C_*i*_ through fragmentation of larger clusters.

The infinite range of cluster sizes, *j* = 1, 2, ..., ∞, in eqs 1 and 2 is an idealization that may be suitable for macroscopic systems as studied in macroscopic experiments. However, in biological cells and in molecular simulations the number of aggregating molecules is limited and the number fluctuations can be significant.^6,7,10,20^ To adapt eqs 1 and 2 to a finite system, one can formally limit the summation ranges, *N*.^19,21,22^ Such truncated Smoluchowski equations have been used to represent the “infinite” Smoluchowski equations (1) as the limit of a finite set of rate equations *N* → ∞.^21^

In this work we assume a finite stochastic model where the probability of an *i*-mer and a *j*-mer to aggregate in the time interval (*t*, *t* + d*t*) is *V*^–1^*K*_*i*,*j*_ dt, where *V* is the system volume and *K*_*i*,*j*_ is the aggregation kernel. The probability that a *k*-mer (with *k* = *i* + *j*) breaks up into an *i*-mer and a *j*-mer in in the time interval (*t*, *t* + d*t*) is *F*_*i*,*j*_ dt, where *F*(*i*, *j*) is the fragmentation kernel *F*_*i*,*j*_. Such stochastic models can be readily simulated numerically using, for instance, the Gillespie algorithm.^23,24^

Starting from the stochastic aggregation–fragmentation model, one can arrive at the following modified Smoluchowski rate equations:
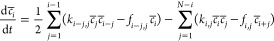
3that describe approximately the time evolution of the average concentrations *c̅*_*i*_, where the average indicated by the bar is taken over realizations of the stochastic aggregation–fragmentation process. We find that the rate constants, *k*_*i*,*j*_ and *f*_*i*,*j*_, and rate kernels, *K*_*i*,*j*_ and *F*_*i*,*j*_, are related as
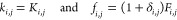
4where the stoichiometric factor 1 + *δ*_*i*,*j*_ accounts for the fragmentation events in which clusters split into equal fragments. Note that in finite systems the average cluster concentrations *c̅*_*i*_ are smooth functions of time and are different from the instantaneous concentrations, *c*_*i*_, that undergo discrete jumps upon aggregation and fragmentation events. Only for large systems, i.e., in the thermodynamic limit, the average concentrations and instantaneous concentrations converge.

The modified Smoluchowski equations (3) are a set of deterministic equations that can be readily solved numerically for small systems. Given a kinetic model specified by the aggregation and fragmentation kernels, *K*(*i*, *j*) and *F*(*i*, *j*), respectively, the modified equations can be used to infer the kinetic parameters and assess the model validity.

In this paper we focus on applications of the modified Smoluchowski equations (3) to the model discrimination and parameter recovery in molecular dynamics simulations of aggregation–fragmentation processes. First, the statistical noise present in small systems is reduced by repeating and averaging the molecular dynamics trajectories. Next, a stochastic model is formulated in terms of the aggregation and fragmenation kernels, *K*_*i*,*j*_ and *F*_*i*,*j*_, and the corresponding modified Smoluchowski equations (3) are fitted to the averages over the simulation repeats. The quality of the fit can be used to discriminate the competing models. Since the modified Smoluchowski equations are an approximate representation of the stochastic process, we need a validation procedure. To this end the recovered kinetic parameters are used as input for the stochastic model that is simulated by a Monte Carlo algorithm. The comparison of the molecular dynamics averages with the Monte Carlo averages is used to validate the model and fitted parameters. We find that, even for small systems, with a number of monomers about *N* = 20, the modified rate equations provide a meaningful description of the averaged aggregation kinetics.

## 2 Methods

In this section we present the stochastic model of aggregation and fragmentation used in this work. General aspects of stochastic kinetics are reviewed in refs (25 and 26). Stochastic models of aggregation–fragmentation have been reviewed in ref (27). Recently, stochastic methods have been applied to study protein self-assembly in small volumes.^7,11,12,20^ Our approach below is quite general and applicable to a diverse range of kinetic schemes that can be characterized by binary aggregation and fragmentation kernels.

### 2.1 Master Equation

Let us consider a homogeneous system that starts at *t* = 0 with *N* monomers in a volume *V*. The monomers move about, collide, and aggregate to form clusters. The clusters may further aggregate to form larger clusters but may also fragment spontaneously into smaller clusters. Here, we consider only binary aggregation and fragmentation events.

The instantaneous state of the systems is determined by the numbers *n*_*i*_, *i* = 1, 2, ..., *N*, of clusters made up of *i* monomers. The total number of free and bound monomers is conserved, ∑_*i*=1_^*N*^ *in*_*i*_ = *N*. The size of the largest possible cluster is *N*.

An *i*-mer, C_*i*_, and a *j*-mer, C_*j*_, may aggregate to form an (*i* + *j*)-mer C_*i*+*j*_, *i* + *j* ≤ *N*:
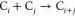
5

A single aggregation event changes the state of the system
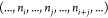
6to
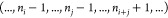
7

For notational convenience, the initial state (6) can be denoted as **n** = (*n*_1_, *n*_2_, ..., *n*_*N*_) and the final state (7) can be denoted as *E*_*i*+*j*_^+^*E*_*j*_^–^*E*_*i*_^–^(**n**), where the step operator *E*_*i*_^±^ acts on any function of **n** as
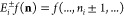
8i.e., changes *n*_*i*_ by 1. A special case emerges when two *i*-mers aggregate:
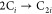
9and change the state (6) to *E*_2*i*_^+^*E*_*i*_^–^*E*_*i*_^–^(**n**):
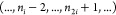
10The probability that an *i*-mer and a *j*-mer aggregate in the time interval (*t*, *t* + d*t*) is *V*^–1^*K*_*i*,*j*_ d*t*, where *K*_*i*,*j*_ is the aggregation kernel.

An (*i* + *j*)-mer, *i* + *j* ≤ *N*, may break into an *i*-mer and a *j*-mer:
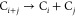
11

A single fragmentation event changes the state (6) into the new state *E*_*i*+*j*_^–^*E*_*j*_^+^*E*_*i*_^+^(**n**):
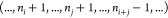
12

A special case is when *i* = *j*, i.e., when a cluster made up of an even number of monomers splits into equal fragments:
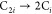
13

The new state is then *E*_2*i*_^–^*E*_*i*_^+^*E*_*i*_^+^(**n**):
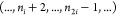
14

The probability that a *k*-mer (with *k* = *i* + *j*) breaks up into an *i*-mer and a *j*-mer in (*t*, *t* + d*t*) is *F*_*i*,*j*_ d*t* where *F*_*i*,*j*_ is the fragmentation kernel.

The aggregation and fragmentation kernels, *K*_*i*,*j*_ and *F*_*i*,*j*_, are symmetric matrices: *K*_*i*,*j*_ = *K*_*j*,*i*_ and *F*_*i*,*j*_ = *F*_*j*,*i*_. Note that a permutation of cluster indexes, (*i*, *j*) → (*j*, *i*), does not introduce a new aggregation or fragmentation processes.

The stochastic system evolution is a sequence of elementary steps (eqs 5 and 11) changing the state of the system. The probability *P*(**n**, *t*) of state **n** at time *t* is governed by the master equation:^26^
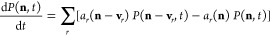
15where **v**_*r*_ is the change in **n** due to a reaction *r* and *a*_*r*_(**n**) is the transition rate of that reaction. The first sum on the rhs is the total entry rate to state **n**, and the second sum is the total exit rate from state **n**.

Aggregation–fragmentation reaction pairs, C_*i*+*j*_ ⇄ C_*i*_ + C_*j*_, can be represented by pairs of indexes *r* = (*i*, *j*) = (*j*, *i*), where *i* + *j* ≤ *N*. The contributions of an aggregation–fragmentation pair *r* = (*i*, *j*) to the total entry rate are
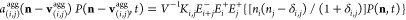
16
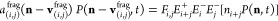
17

The contributions of the (*i*, *j*) aggregation–fragmentation reactions to the exit rate are
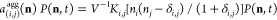
18
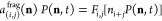
19

The aggregation and fragmentation kernels, *K*_*i*,*j*_ and *F*_*i*,*j*_, determine the stochastic kinetics as expressed in terms of the master equation (15).

In finite systems, the instantaneous concentrations, *c*_*i*_ = *n*_*i*_/*V*, take discrete values. Here, we are interested in the averages, *c̅*_*i*_(*t*), of the instantaneous cluster concentrations over simulation repeats:
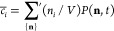
20where the prime indicates the summation over all states satisfying ∑_*i*=1_^*N*^ *in*_*i*_ = *N*, and the bar indicates the average over an ensemble of trajectories.

### 2.2 Gillespie Algorithm

In this work we use the direct Gillespie stochastic simulation algorithm^23,24^ to generate trajectories of the stochastic process represented by the master equation (15). In each step two random numbers *u*_1_ and *u*_2_ are drawn from the uniform distribution in the unit interval. The time *t* and species populations **n** = (*n*_1_, *n*_2_, ..., *n*_*N*_) are updated by randomly sampling the waiting time and selecting the reaction that occurs in that step.

The simulation is initiated by setting the species populations to their initial values. In our case, only monomers are present at *t* = 0 so *n*_*i*_ = *N*δ_1,*i*_. The time interval τ to the next reaction is then drawn from the exponential distribution as
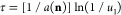
21where
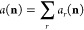
22is the sum of the rates *a*_*r*_ of all potential reactions as determined by the current species populations **n**. The index of the next reaction to execute is drawn as the smallest integer *j* satisfying
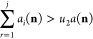
23

The selected reaction *r* is then executed by replacing **n** ← **n** + **v**_*r*_, where **v**_*r*_ is the change in **n** due to reaction *r*. Time is updated by replacing *t* ← *t* + τ. This process generates a single trajectory and may be repeated and averaged.

In this work we ran the direct Gillespie algorithm using an adaptation of the software made available at Github by Sauro, https://github.com/sys-bio/libStochastic.^28^

### 2.3 Modified Smoluchowski Rate Equations

Let us consider the mean number of cluster of size *i*, ⟨*n*_*i*_⟩. Upon taking the appropriate sums in (15), one finds the first equation in a hierarchy of coupled moments:
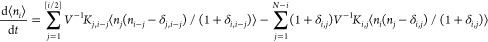
24
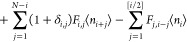
25where [*x*] is the integer part of *x*. A different way of writing this equation is
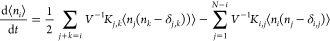
26
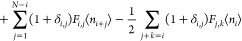
27where the factor 1/2 prevents double-counting.

The modified Smoluchowski rate equations that account for the finite system size and aggregation reversibility are obtained from ⟨*n*_*i*_(*n*_*j*_ – *δ*_*i*,*j*_⟩ ≈ ⟨*n*_*i*_⟩⟨*n*_*j*_⟩, i.e., by assuming that the *n*_*i*_ and *n*_*j*_ are uncorrelated and that *n*_*j*_ – 1 ≈ *n*_*j*_. Since *c̅*_*i*_ = ⟨*n*_*i*_⟩/*V*, one gets
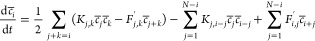
28where the prime indicates the modified fragmentation coefficient
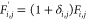
29

The rate equations (28) and (29) are equivalent to (3) and (4).

### 2.4 Connection with Chemical Kinetics

The rate equations for species concentrations are ordinary differential equations that may take different numerical coefficients depending on the definition of the rate coefficients and on whether the stoichiometric coefficients are expressed explicitly or implicitly. We adopted the conventional forms in eqs 1–3 for their simplicity and clear interpretation. For clarity of exposition, we briefly discuss the connection between the rate equations, rate kernels, rate coefficients, and stoichiometric factors.

The primary quantities in the stochastic kinetics of aggregation and fragmentation are the rate kernels *K*_*i*,*j*_ and *F*_*i*,*j*_. The quantity *V*^–1^*K*_*i*,*j*_ is the probability per unit time that a given pair of clusters of sizes *i* and *j* will aggregate in a volume *V*. The number of available *i*-mer and *j*-mer pairs is *n*_*i*_*n*_*j*_ when *i* ≠ *j* and *n*_*i*_(*n*_*i*_ – 1)/2 when *i* = *j*, which can be written compactly as *n*_*i*_(*n*_*j*_ – *δ*_*i*,*j*_)/(1 + *δ*_*i*,*j*_). The rate of aggregation events, i.e., the number of aggregation events per unit time, is *V*^–1^*K*_*i*,*j*_*n*_*i*_(*n*_*j*_ – *δ*_*i*,*j*_)/(1 + *δ*_*i*,*j*_), which we approximate as *V*^–1^*K*_*i*,*j*_*n*_*i*_*n*_*j*_/(1 + *δ*_*i*,*j*_). The rate of aggregation events per unit volume is thus *K*_*i*,*j*_*c*_*i*_*c*_*j*_/(1 + *δ*_*i*,*j*_), where *c*_*i*_ and *c*_*j*_ are the concentrations of *i*-mers and *j*-mers, respectively. Note that the classical chemical kinetics uses a convention that the rate of a bimolecular reaction is written as *k*_*i*,*j*_^′^*c*_*i*_*c*_*j*_ or *k*_*i*_^′^*c*_*i*_^2^. The rate equations for the cluster concentrations account for the stoichiometric coefficients of binary aggregation, i.e., the number of *i*-mers that disappear or are generated in a single event. The special case is when two equal-size clusters aggregate, C_*i*_ + C_*i*_ → C_2*i*_, or a cluster with an even number of particles splits into identical fragments, C_2*i*_ → C_*i*_ + C_*i*_.

Similarly, the fragmentation kernel *F*_*i*,*j*_ is the probability per unit time that a given cluster of size *i* + *j* will fragment into a pair of clusters with sizes *i* and *j*. The rate of fragmentation events of clusters C_*i*+*j*_ is *F*_*i*,*j*_*n*_*i*+*j*_. The rate of fragmentation events per unit volume is *F*_*i*,*j*_*c*_*i*+*j*_. When *i* = *j*, two particles C_*i*_ are created from C_2*i*_ in one event. The classical chemical kinetics writes the rate of fragmentation as *f*_*i*,*j*_^′^*c*_*i*+*j*_, and an explicit stoichiometric factor appears in the rate equations. In eqs 2 and 3 the stoichiometric coefficients are adsorbed into the rate coefficients *f*_*i*,*j*_ as in eq 4.

### 2.5 Model Aggregation and Fragmentation Kernels

Below we consider examples of aggregation–fragmentation kernels that are used in the next sections.

When the aggregation kernel does not depend on the cluster sizes
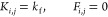
30the Smoluchowski equations (1) can be solved analytically for monodisperse initial conditions, *c*_*i*_(0) = *cδ*_1*i*_:^18^
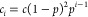
31where
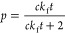
32and *c* is the initial concentration of monomers.

Analytical expressions for the *c*_*i*_(*t*) are known for some size dependent rate constants, for instance, for additive kernel:
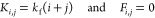
33and the monodisperse initial conditions, *c*_*i*_(0) = *cδ*_1*i*_:^18^
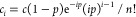
34where
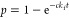
35

The aggregation kernel derived by Smoluchowski^17^ for diffusing and coalescing particles can be written as
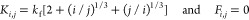
36where *k*_f_ is a composite constant.

Blatz and Tobolsky^29^ considered the model of reversible aggregation that, in the present notation, can be defined as
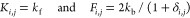
37

This model can be solved as
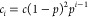
38where
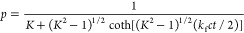
39and
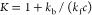
40

The functional form of a realistic fragmentation kernel is difficult to obtain as the fragmentation rate and the distribution of fragments depend upon many microscopic details such as aggregate morphology and connectivity. To account for the size dependent aggregation–fragmentation in our molecular dynamics data (see Results and Discussion), we used the following three-parameter model:
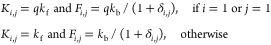
41where the aggregation and fragmentation of monomers are faster by a factor of *q*.

## 3 Results and Discussion

In this section we consider examples illustrating the application of the modified Smoluchowski equations (3) for kinetic modeling. First, we discuss three stochastic models for aggregation in finite systems, *N* = 5 and *N* = 20, and show that the modified Smoluchowski equations can provide a good approximation for the average concentrations even for small systems, *N* = 20. Second, we consider the aggregation kinetics of a coarse-grained molecular dynamics model that reproduces the various morphologies of peptide aggregates.^30^ We show how our approach can be used to develop a kinetic model that describes the initial aggregation–fragmentation kinetics. Third, we consider the aggregation kinetics of a coarse-grained model of a short polyglutamine peptide (Q12). We simulated the aggregation at two concentrations and found that single-concentration fits are satisfactory, but the global kinetic fits, attempting to link parameters over two different concentrations, fail. This example illustrates the limitations of the stochastic aggregation kinetics underlying the modified Smoluchowski equations.

### 3.1 Stochastic Kinetics

**Figure 1:**
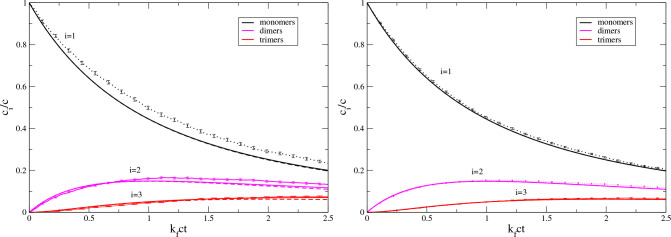
Scaled aggregation kinetics, *c*_*i*_/*c* vs *k*_f_*ct*, of the monomers, *i* = 1, dimers, *i* = 2, and, trimers, *i* = 3, for the Smoluchowski irreversible aggregation model, eq 30. The initial numbers of free monomers are *N* = 5 (left panel) and *N* = 20 (right panel). The averages for 1000 repeats of stochastic simulations are shown as dotted lines with error bars indicating the standard deviation of the mean. The solutions of the modified Smoluchowski equations (3) are presented as solid lines. The stochastic simulations and the modified Smoluchowski approach converge as *N* increases. The deterministic limit, *N* = ∞, is shown as broken lines.

In this subsection we compare the simulated stochastic kinetics with that calculated from the modified Smoluchowski equations (3). We start with the Smoluchowski irreversible aggregation model; see Figure 1. We simulated 1000 repeats of the aggregation kinetics for *N* = 5 and *N* = 20 monomers using the Gillespie algorithm^23,24^ and calculated the time evolutions of average concentrations for monomers, dimers, and trimers. Figure 1 demonstrates that for a small system with *N* = 5 monomers the solutions of the modified Smoluchowski equations deviate from the averaged simulations, but those deviations disappear as the system gets larger, *N* = 20. Interestingly, the modified Smoluchowski kinetics for small oligomers and short times are close to the exact deterministic result for *N* = ∞.

**Figure 2:**
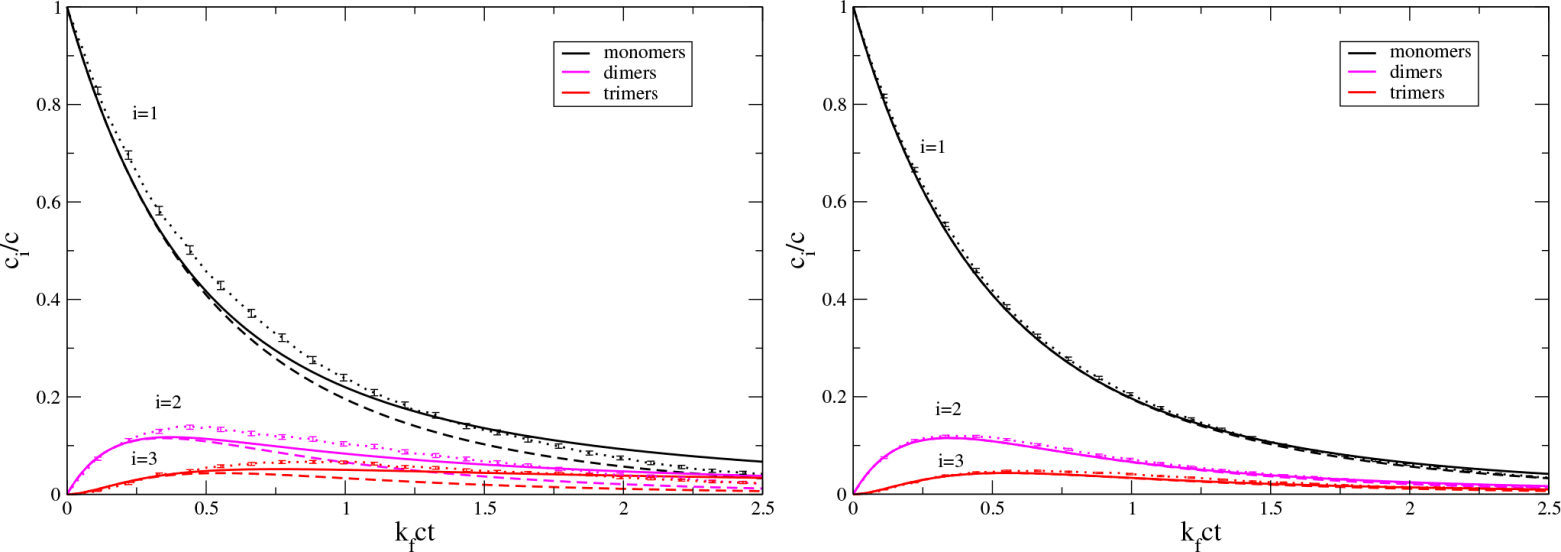
Same as in Figure 1 but for the additive aggregation kernel, eq 34.

A similar behavior can be seen for the additive aggregation kernel as shown in Figure 2. Note, however, that the modified Smoluchowski kinetics for small oligomers and short times are not as close to the exact deterministic result for *N* = ∞ as for the Smoluchowski model.

**Figure 3:**
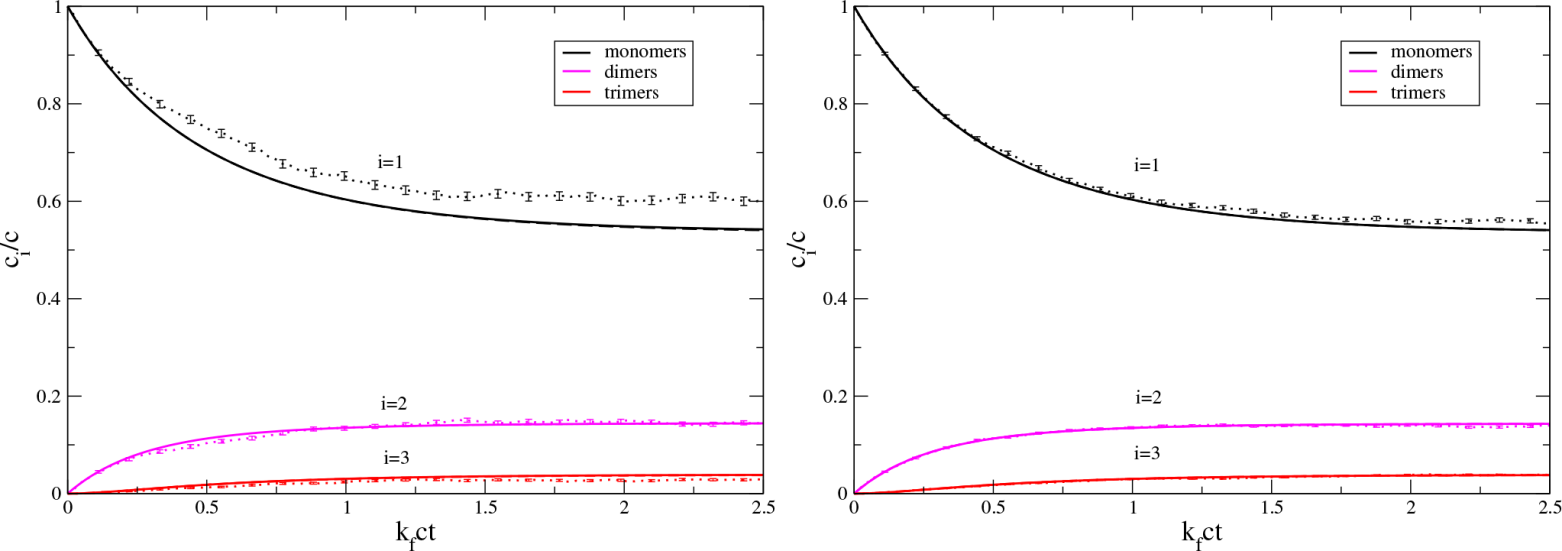
Same as Figure 1 but for the Blatz–Tobolsky aggregation–fragmentation model, eq 37, with *k*_b_/(*k*_f_*c*) = 1.

Our third stochastic kinetics example deals with the Blatz–Tobolski model of aggregation–fragmentation kinetics; see Figure 3. This is another example that the modified Smoluchowski equations (3) can be a good approximation to the “true” stochastic kinetics for systems as small as *N* = 20.

**Figure 4:**
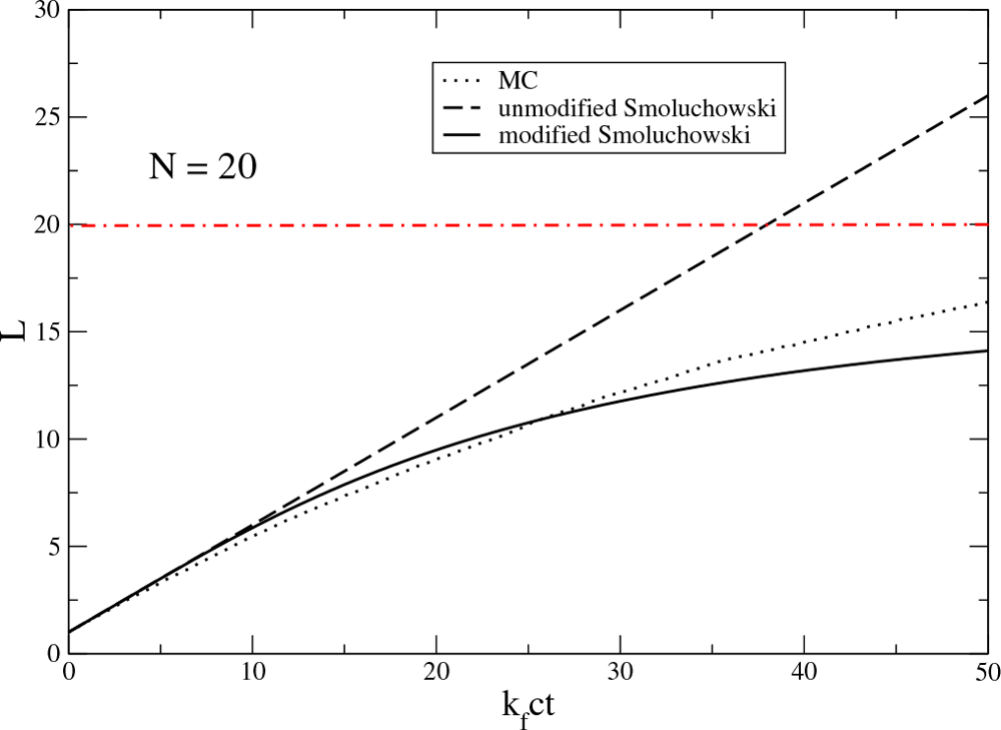
Number-average aggregate size *L* as a function of the scaled time *k*_f_*ct* for the Smoluchowski irreversible aggregation model, eq 30. The initial number of free monomers is *N* = 20. The average *L* for 1000 repeats of the stochastic Monte Carlo (MC) simulations is shown as a dotted line. The solution of the modified Smoluchowski equation (3) is presented as a solid line. The deterministic limit (unmodified Smoluchowski) is shown as a broken line. The horizontal line at *L* = 20 shows the maximum aggregate size.

In the above examples, the modified Smoluchowski kinetics for small oligomers and short times are close to the exact deterministic result for *N* = ∞ and to the “true” averaged stochastic kinetics; see the right panels in Figures 1–3. This suggests that the unmodified Smoluchowski kinetics might be used to fit the kinetic data. This is indeed the case for the above kinetic schemes, where the analytical solutions are known. For instance, the unmodified irreversible Smoluchowski model was used to rationalize the molecular dynamics data in ref (16). However, such an approach is limited to early times when the number of kinetic units, *V*∑_*i*_ *c*_*i*_(*t*), in the system volume *V* is greater than 1. At long times the number of kinetic units in any finite volume becomes less than 1, which may lead to unphysical predictions. For instance, as shown in Figure 4, at longer times the unmodified Smoluchowski kinetics predicts that the average cluster size is larger than the system size *N*. The modified Smoluchowski equations are free of this shortcoming.

An additional consideration is that the exact solutions to the Smoluchowski kinetics are in general not known, in particular for the reversible case. Thus one needs to resort to numerical solutions which require a truncation to a finite number of equations that are numerically tractable, as for example in ref (15). The modified Smoluchowski equations provide such a consistent truncation for finite systems. Thus we argue that the modified Smoluchowski equations may be a practical tool for fitting the kinetics in finite systems.

### 3.2 Discrimination of Molecular Dynamics Kinetic Models

Now we demostrate how the modified Smoluchowski equations (3) can be used to infer the kinetic model from molecular dynamics simulations of a small system. We consider a coarse-grained two-beads-per-residue model that reproduces diverse morphologies of the aggregates.^30^ In this homopeptide model each residue is represented by two beads: one for the backbone and one for the side chain. The beads interact via the standard Lennard-Jones (LJ) potential. Additionally, two pseudocharged beads cap the peptide termini. The termini beads interact via the LJ potentials with interaction strengths corresponding to the repulsion and attraction between the like or oppositely charged ends, respectively. A detailed model description is available in our previous paper.^30^

We used the GROMACS 2020.5 package^31^ for the simulation and MDAnalysis^32^ together with python homemade scripts for data analysis. The periodic boundary conditions were used in all dimensions. After an initial energy minimization, production simulations were run with an *NVT* ensamble with the leapfrog stochastic dynamic integrator serving as a thermostat at 303 K. The 1 μs simulations starting with *N* = 72 monomers were repeated three times. A distance criterion was used to detect aggregation–fragmentation events with a cutoff of 0.55 nm. From our simulations, we extracted the time evolutions of the average numbers of monomers, dimers, and trimers and the total number of clusters including monomers and used those quantities to develop a kinetic model.

Equation 3 was solved numerically for the average numbers of oligomers *n̅*_*i*_ = *c̅*_*i*_*V*. The total number of clusters was calculated as *n*_cluster_ = ∑_*i*=1_^*N*^ *n*_*i*_. The numbers of monomers, dimers, and trimers and the total number of clusters including monomers were globally fit to the averaged simulation curves by minimizing the scaled sum of the weighted squared differences between the simulation data and model. For each set of the fit parameters, we generated 1000 stochastic repetitions of the aggregation–fragmentation kinetics using the Gillespie algorithm^23,24^ and found that, in the present case of *N* = 72 monomers, the averaged stochastic kinetics is well represented by the modified Smoluchowski equations.

**Figure 5:**
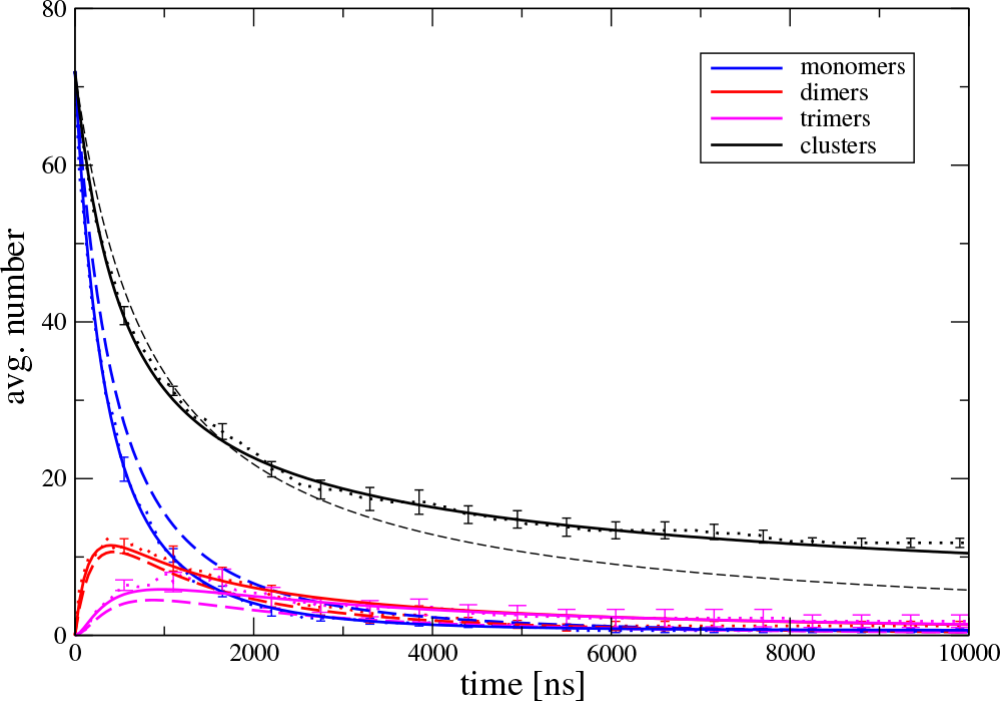
Aggregation kinetics of two-beads-per-residue molecular dynamics model, eq 41. The average numbers of monomers, dimers, and trimers and the total number of clusters including monomers are shown as dotted lines with error bars indicating the standard deviation of the mean. The broken lines indicate the best fit for the Smoluchowski model, eq 30, and the solid lines indicate the fit to the three-parameter model, eq 41.

Figure 5 and Table 1 illustrate the development of the kinetic model. The minimized fit function was the reduced chi-square statistic, χ_ν_^2^, defined as the ratio χ^2^/ν, where χ^2^ is the weighted sum of squared deviations between the data and model and ν = *n* – *m* is the number of degrees of freedom, i.e., the number of observations *n* minus the number of fitted parameters *m*. The reduced chi-square allows for comparison of models with different numbers of parameters *m*. A value of χ_ν_^2^ close to 1 indicates a good fit.

**Table 1:** Reduced Chi-Square, *χ*_ν_^2^, for the Models Fitted to the Molecular Dynamics Data in Figure 5^a^

model	one param, eq 30	one param, eq 36	two params, eq 37	three params, eq 41
χ_ν_^2^	10.85	10.85	10.85	0.99

a The column heads show the number of fitted parameters in a model. Only a three-parameter fit gave satisfactory results.

We began our kinetic analysis with the modified Smoluchowski model assuming irreversible aggregation with size independent rate constants; see the eq 30 entry in Table 1. The best fit for the Smoluchowski model is shown as the broken line in Figure 5. Clearly, this model does not describe well the molecular dynamics aggregation kinetics. Then, we tried other one-parameter irreversible models but did not get any improvement. For instance, the irreversible diffusion model, eq 36, gave a fit hardly distinguishable from the Smoluchowski model. We took this as an indication that fragmentation should be included. We tried the two-parameter Blatz–Tobolsky model, eq 37, but the fits of this model converged to *k*_b_ = 0, i.e., reproduced the Smoluchowski model fits. The fits of the irreversible diffusion model and Blatz–Tobolsky model were indistinguishable from the broken line in Figure 5. This suggested that a size dependent kernel is needed. To limit the number of fitted parameters, we assumed a three-parameter model with a minimal change compared to the Blatz–Tobolsky model, eq 41, that satisfactorily reproduces the molecular dynamics data.

### 3.3 Applicability of Stochastic Models for Molecular Kinetics

In the above examples we have considered the aggregation–fragmentation kinetics for a single initial concentration *c*. Now we compare the kinetics at two initial concentrations, *c*_A_ and *c*_B_. Specifically, we consider the molecular dynamics kinetics of 12-residue polyglutamine, Q12, in the coarse-grained explicit solvent SIRAH force field.^33,34^ This force field maps the backbone chains into nitrogen, α-carbon, and oxygen beads, while side chains are modeled at a lower resolution. Water is represented by four linked beads, each carrying a partial charge.

We compare our Q12 simulation data for systems with *N* = 20 and *N* = 50 peptides at two concentrations, *c*_A_ = 10 mM and *c*_B_ = 20 mM, in the presence of 0.2 mM NaCl. The GROMACS 2018.8 package was used for the simulations and data analysis.^31^ The periodic boundary conditions were used in all dimensions. After an initial energy minimization, production simulations were run with the Rahman–Parrinello barostat at 1 bar and the V-rescale thermostat at 300 K. The 1 μs simulations were repeated six times for each concentration when *N* = 20 and three times when *N* = 50. A distance criterion was applied for molecular aggregation events with a cutoff distance of 0.6 nm.

**Figure 6:**
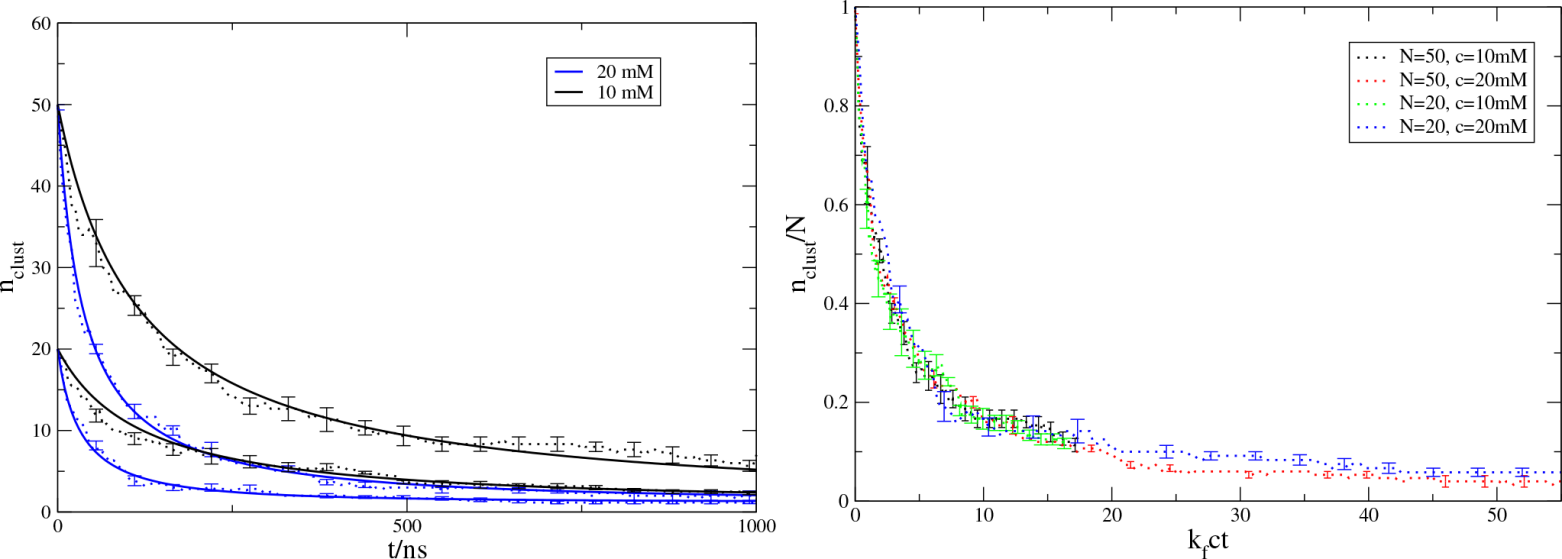
Aggregation kinetics of a coarse-grained molecular dynamics model of polyglutamine, Q12, at two concentrations, *c*_A_ = 10 mM and *c*_B_ = 20 mM, and two initial numbers of free monomers, *N* = 20 and *N* = 50. (left) The average numbers of clusters are shown as dotted lines with error bars indicating the standard deviation of the mean. The solid lines show the best fit for the Smoluchowski model (30). (right) Scaled kinetics, *c*_*i*_/*c* vs *k*_f_*ct*, for the data in the left panel. The scaled curves collapse approximately onto a master curve.

The Smoluchowski irreversible aggregation model could be fit to the time evolution of *n̅*_cluster_ at individual concentrations; see Figure 6. Table 2 shows the fitted rate constants. The fits for *N* = 20 and *N* = 50 are consistent for each concentration, so the stochastic model describes well the dynamics for individual concentrations. However, *k*_f_ changes significantly when the concentration changes. The underlying stochastic model assumes that the rate kernels are independent of the concentration. We conclude that, in this particular case, the stochastic model is not adequate to describe the global aggregation kinetics when the data across concentrations are combined. We speculate that the origin of this discrepancy is the internal restructuring of the clusters that is not accounted for in the current stochastic model.

**Table 2:** Fitted Rate Coefficient *k*_f_ of the Irreversible Smoluchowski Model, *K*(*i*, *j*) = *k*_f_ and *F*(*i*, *j*) = 0, for Two Initial Concentrations, *c*, and Two Numbers of Monomers, *N*

*c*/mM	*N*	*k*_f_/(nm^3^ ps^–1^)	χ_ν_^2^^a^
10	20	2.73	1.20
	50	2.87	1.54
20	20	5.23	1.24
	50	4.63	1.69

a Quality of the fits as the final value of the minimized reduced chi-square function.

## 4 Conclusion

In molecular dynamics simulations, the computation cost limits the system size, the simulation length, and the number of simulation repeats. Our goal was to explore whether the modified Smoluchowski equations (3) can be used to distinguish kinetic models and infer their parameters. We justified the applicability of eq 3 using stochastic simulations of the corresponding master equation.

The modified Smoluchowski rate equations bring two major modifications with respect to the Smoluchowski theory (2). First, they deal with a finite, rather than infinite, number of linked rate equations, which corresponds to the finite number of aggregating monomers. Second, they deal with the average concentrations, rather than instantaneous concentrations. The modified Smoluchowski equations provide an approximation to the time evolutions of the average species numbers and may fail for small systems. When the system becomes large, the modified rate equations converge to the “infinite” Smoluchowski model.

We considered several examples of applications of the modified Smoluchowski equations (3). We focused on the use of the modified Smoluchowski equations for interpreting the kinetics in molecular dynamics simulations. Our examples demonstrated eq 3 provides a good approximation for the average kinetics stochastic dynamics even for small systems, e.g., *N* = 20 monomers.

Molecular aggregation and fragmentation in finite systems are intrinsically stochastic processes. In this work, we assumed that their aggregation kinetics can be described by the stochastic aggregation–fragmentation master equation as defined in eq 15. We showed that a simple binary aggregation–fragmentation model may fail when the kinetics for two initial concentrations are analyzed.

The main conclusion of this work is that the modified Smoluchowski equations (3) combined with Monte Carlo simulations of the corresponding master equation provide a practical and effective tool for developing kinetic models of peptide aggregation in molecular dynamics simulations.
